# Histone Deacetylase Inhibitors Downregulate Calcium Pyrophosphate Crystal Formation in Human Articular Chondrocytes

**DOI:** 10.3390/ijms23052604

**Published:** 2022-02-26

**Authors:** Chi-Ching Chang, Kun-Lin Lee, Tze-Sian Chan, Chia-Chen Chung, Yu-Chih Liang

**Affiliations:** 1 Division of Allergy, Immunology and Rheumatology, Department of Internal Medicine, School of Medicine, College of Medicine, Taipei Medical University, Taipei 11031, Taiwan; ccchang@tmu.edu.tw; 2 Division of Rheumatology, Immunology and Allergy, Department of Internal Medicine, Taipei Medical University Hospital, Taipei 11031, Taiwan; 3 School of Medical Laboratory Science and Biotechnology, College of Medical Science and Technology, Taipei Medical University, Taipei 11031, Taiwan; kunlinleetw@gmail.com (K.-L.L.); shettoangel@gmail.com (C.-C.C.); 4 Ph.D. Program in Medical Biotechnology, College of Medical Science and Technology, Taipei Medical University, Taipei 11031, Taiwan; 5 Division of Gastroenterology, Department of Internal Medicine, School of Medicine, College of Medicine, Taipei Medical University, Taipei 11031, Taiwan; fzesian@tmu.edu.tw; 6 Division of Gastroenterology, Department of Internal Medicine, Wan Fang Hospital, Taipei 11696, Taiwan; 7 Ph.D. Program in Drug Discovery and Development Industry, College of Pharmacy, Taipei Medical University, Taipei 11031, Taiwan; 8 Traditional Herbal Medicine Research Center, Taipei Medical University Hospital, Taipei 11031, Taiwan

**Keywords:** calcium pyrophosphate, HDAC, ANKH, ENPP1, TNAP

## Abstract

Calcium pyrophosphate (CPP) deposition disease (CPPD) is a form of CPP crystal-induced arthritis. A high concentration of extracellular pyrophosphate (ePPi) in synovial fluid is positively correlated with the formation of CPP crystals, and ePPi can be upregulated by ankylosis human (ANKH) and ectonucleotide pyrophosphatase 1 (ENPP1) and downregulated by tissue non-specific alkaline phosphatase (TNAP). However, there is currently no drug that eliminates CPP crystals. We explored the effects of the histone deacetylase (HDAC) inhibitors (HDACis) trichostatin A (TSA) and vorinostat (SAHA) on CPP formation. Transforming growth factor (TGF)-β1-treated human primary cultured articular chondrocytes (HC-a cells) were used to increase ePPi and CPP formation, which were determined by pyrophosphate assay and CPP crystal staining assay, respectively. Artificial substrates thymidine 5′-monophosphate p-nitrophenyl ester (p-NpTMP) and p-nitrophenyl phosphate (p-NPP) were used to estimate ENPP1 and TNAP activities, respectively. The HDACis TSA and SAHA significantly reduced mRNA and protein expressions of ANKH and ENPP1 but increased TNAP expression in a dose-dependent manner in HC-a cells. Further results demonstrated that TSA and SAHA decreased ENPP1 activity, increased TNAP activity, and limited levels of ePPi and CPP. As expected, both TSA and SAHA significantly increased the acetylation of histones 3 and 4 but failed to block Smad-2 phosphorylation induced by TGF-β1. These results suggest that HDACis prevented the formation of CPP by regulating ANKH, ENPP1, and TNAP expressions and can possibly be developed as a potential drug to treat or prevent CPPD.

## 1. Introduction

Calcium pyrophosphate (CPP) deposition disease (CPPD) is a manifestation of the abnormal accumulation of CPP crystals in a joint. The main risk factor for CPPD is aging, and 75% of acute patients are aged over 85 years [1]. Symptoms of CPPD include inflammation and cartilage degeneration, which are similar to a gout attack; therefore, this condition is also called pseudogout [2]. Extracellular pyrophosphate (ePPi), calcium, and extracellular matrix (ECM) are essential components of CPP crystal formation [3]. In CPPD patients, a high concentration of ePPi in the synovial fluid is mainly locally produced in joints [4]. Many diseases are characterized by high levels of ePPi in synovial fluid, including osteoarthritis (OA) with CPPD [5], hyperparathyroidism [6], hypomagnesemia [7], and hemochromatosis [8]. The ePPi in synovial fluid originates from ATP hydrolysis by ectonucleotide pyrophosphatase 1 (ENPP1, also known as phosphodiesterase family member 1 (PC-1)) [9,10] or efflux from the cytosol by the diphosphate transmembrane transporter, ANKH [11]. Otherwise, ePPi can be cleaved into phosphate by tissue non-specific alkaline phosphatase (TNAP, also called alkaline phosphatase, liver (ALPL)) [12].

Previous studies found that mutations or single-nucleotide polymorphisms of the *ANKH* gene may increase the function of ANKH, leading to increased ePPi levels and CPPD incidence [13]. In addition to efflux of PPi, ANKH exports ATP to the extracellular space, which may increase the cleavage of ATP into PPi [14]. The concentration of ePPi is mainly regulated by several growth factors and cytokines, including transforming growth factor (TGF)-β [13,15], interleukin (IL)-1β [16], and insulin-like growth factor (IGF)-1 [14]. Other factors were also found to promote the formation of CPP crystals, such as type 2 transglutaminase [17,18], coagulation factor XIIIa [19], and osteopontin [20]. TGF-β increases the activities of PC-1, ANKH, cartilage intermediate layer protein (CILP), and transglutaminase and reduces the activity of TNAP, ultimately upregulating the level of ePPi [13,15]. Conversely, IGF-1 and inflammatory cytokines (such as IL-1β) can lead to lower ePPi levels [14].

In epigenetics, histone acetylation and DNA methylation were shown to play key roles in regulating gene expressions and cell functions [21]. Two different groups of enzymes, namely histone acetyltransferases (HATs) and histone deacetylases (HDACs), are responsible for regulation of acetylation and deacetylation, respectively [22]. Besides histones, some non-histone proteins can also be modified by HDACs [23,24]. The acetylation of various kinds of proteins was shown to play roles in a variety of cellular processes, such as gene expression, chromatin remodeling, splicing, nuclear transport, cell cycle progression, actin polymerization, and DNA damage repair, as well as the functions of the cytoskeleton, chaperones, and ribosomes [25].

HDAC inhibitors (HDACis) accelerate osteoblast differentiation but reduce osteoclastogenesis [26]. Studies have also confirmed that HDAC plays a critical role in maintaining the balance between osteoblast bone formation and osteoclast bone resorption [27]. HDACis can promote the process of osteogenic differentiation, expressions of osteoblast-related genes, and the formation of bone nodules [28]. In a mouse experiment of skull defects, pre-osteoblasts pretreated with an HDACi exhibited enhanced bone regeneration [29], which demonstrated that HDAC is an important epigenetic factor driving mineral tissue regeneration.

In macrophages, CPP crystals can activate the inflammasome via the NLR family pyrin domain-containing 3 (NLRP3) and induce the production of the pro-inflammatory factor IL-1β, which in turn stimulates the production of other cytokines, such as tumor necrosis factor (TNF)-α to amplify the inflammatory response [30]. CPP crystals can also cause nitric oxide (NO) production through the Toll-like receptor 2 (TLR2) pathway in chondrocytes [31]. In addition, CPP crystals inhibit neutrophil apoptosis and stimulate neutrophils to release myeloperoxidase, IL-1β, IL-8, IL-6, and neutrophil extracellular traps (NETs) and then cause damage to bystander cells and tissues [32].

At present, many treatments are available to improve symptoms, but they cannot cure CPPD; these include colchicine, hydroxychloroquine, non-steroidal anti-inflammatory drugs (NSAIDs), corticosteroids, and anakinra [33,34,35,36]. Once CPP crystals are deposited, they can only be removed by drawing out the synovial fluid. Therefore, preventing CPP crystal formation is a preferable strategy against CPPD. In this study, we investigated the roles of the HDACis trichostatin A (TSA) and vorinostat (SAHA) in preventing CPP crystal formation using primary cultured human articular chondrocytes (HC-a cells) in a TGF-β1 model. We found that both TSA and SAHA decreased CPP formation by downregulating *ENPP1* and *ANKH* and upregulating *TNAP* gene expressions independent of the TGF-β1-induced Smad signal pathway.

## 2. Results

### 2.1. HDACis Downregulated ANKH and ENPP1 Expressions and Upregulated TNAP Expression

**Figure 1:**
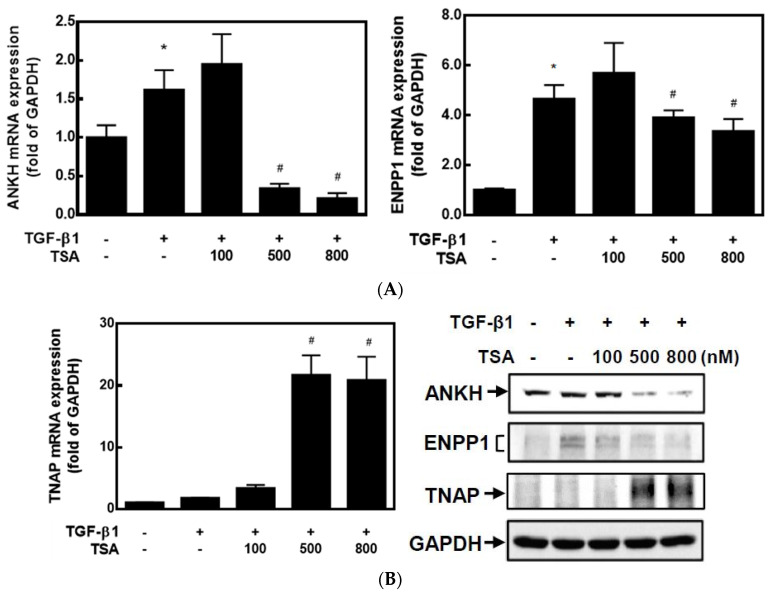
Trichostatin A (TSA) changed the calcium pyrophosphate (CPP)-related gene expressions in transforming growth factor (TGF)-β1-treated chondrocytes. Human primary chondrocytes (HC-a cells) were pretreated with different concentrations of TSA (100–800 nM) for 30 min and then treated with 5 ng/mL TGF-β1 for 24 h. (**A**) Total RNAs were extracted, and mRNA expression levels of *ANKH*, *ENPP1*, and *TNAP* were determined by real-time PCR. Data are expressed as the mean ± SE of five independent experiments. * *p* < 0.05 vs. column 1; # *p* < 0.05 vs. column 2. (**B**) Total cellular proteins were collected, and protein expressions were detected by Western blotting.

**Figure 2:**
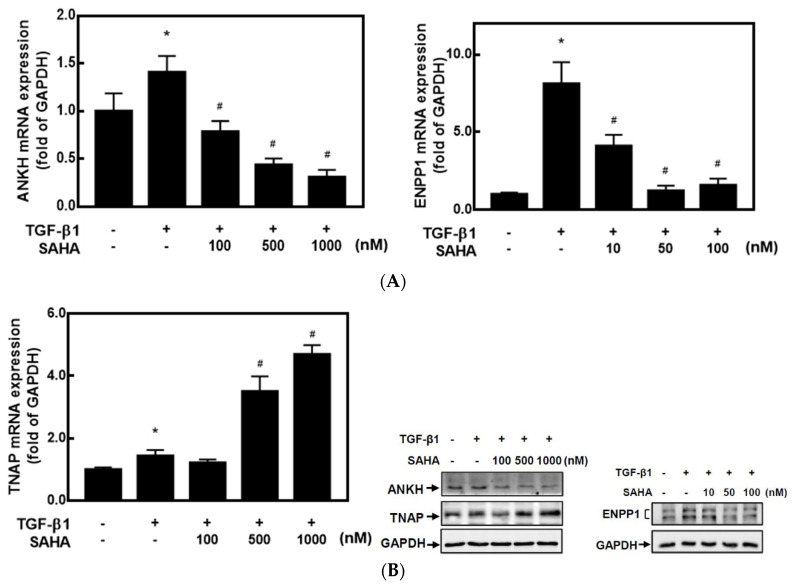
Vorinostat (SAHA) changed calcium pyrophosphate (CPP)-related gene expressions in transforming growth factor (TGF)-β1-treated chondrocytes. Human primary chondrocytes (HC-a cells) were pretreated with different concentrations of SAHA (10–1000 nM) for 30 min and then treated with 5 ng/mL TGF-β1 for 24 h. (**A**) Total RNAs were extracted, and mRNA expression levels of *ANKH*, *ENPP1*, and *TNAP* were determined by real-time PCR. Data are expressed as the mean ± SE of at least four independent experiments. * *p* < 0.05 vs. column 1; # *p* < 0.05 vs. column 2. (**B**) Total cellular proteins were collected, and protein expressions were detected by Western blotting.

It is known that HDACis can regulate gene expressions by changing epigenetic modifications. To understand whether HDACis can regulate CPPD-related gene expressions, we used primary cultured human articular chondrocytes (HC-a cells) and two HDACis, TSA and SAHA. As mentioned in the Introduction, high levels of ANKH and ENPP1 promote CPP crystal formation, whereas TNAP reduces CPP crystal formation. TSA significantly decreased mRNA expressions of *ANKH* and *ENPP1* but increased *TNAP* mRNA expression in dose-dependent manners compared to TGF-β1-treated cells (Figure 1A). Western blot analysis also demonstrated that TSA dose-dependently inhibited protein expressions of ANKH and ENPP1 but increased TNAP expression (Figure 1B). On the other hand, SAHA exhibited similar trends of inhibiting both *ANKH* and *ENPP1* expression and increasing *TNAP* expression. Compared to TGF-β1 treatment, 100–1000 nM of SAHA significantly decreased *ANKH* and increased *ENPP1* mRNA expression, but lower concentrations of SAHA (10–100 nM) inhibited *ENPP1* mRNA expression (Figure 2A). Again, a similar trend of protein levels was found by a Western blot analysis (Figure 2B). These results suggest that both HDACis, TSA and SAHA, were able to inhibit expression of ANKH and ENPP1 but increased TNAP expression.

### 2.2. HDACis Significantly Downregulated ENPP1 and Upregulated TNAP Activities in HC-a Cells

**Figure 3:**
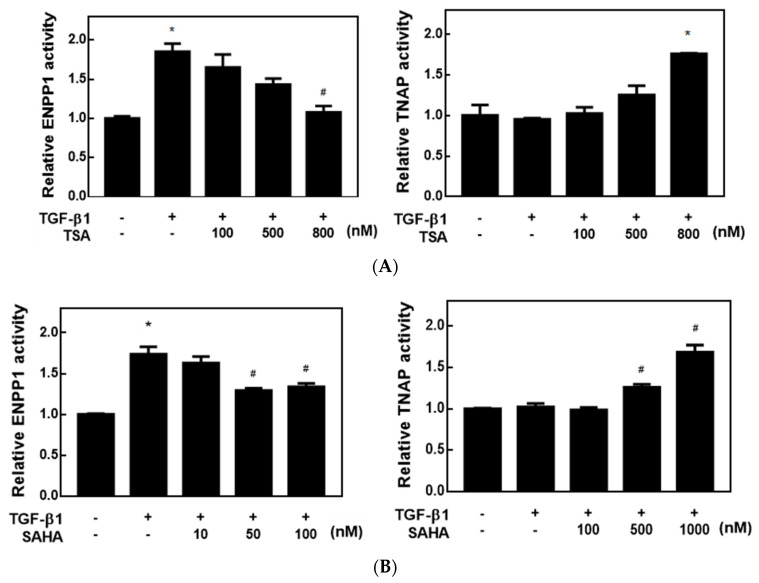
Histone deacetylase inhibitors (HDACis) decreased ectonucleotide pyrophosphatase 1 (ENPP1) activity and increased tissue non-specific alkaline phosphatase (TNAP) activity in transforming growth factor (TGF)-β1-treated chondrocytes. (**A**,**B**) Human primary chondrocytes (HC-a cells) were pretreated with different concentrations of trichostatin A (TSA) (100~800 nM) and vorinostat (SAHA) (100–1000 nM) for 30 min and then treated with 5 ng/mL TGF-β1 for 48 h. Total cellular lysates were collected to determine ENPP1 and TNAP activities as described in Materials and Methods. Data are expressed as the mean ± SE of at least three independent experiments. * *p* < 0.05 vs. column 1; # *p* < 0.05 vs. column 2.

Since HDACis can regulate CPP-related gene and protein expressions, we next examined whether HDACis could change CPP-related enzyme activities in cells using two artificial substrates: p-NpTMP for ENPP1 activity and p-NPP for TNAP activity. Both enzyme reactions produce the same end product, called p-NP (p-nitrophenol), which can be detected at OD410 nm by a spectrophotometer. HC-a cells were treated with different concentrations of HDACis for 24 h, and then total cell lysates were collected to determine the cellular enzyme activities of ENPP1 and TNAP. As shown in Figure 3, TSA dose-dependently downregulated ENPP1 activity and upregulated TNAP activity in a cell assay. In addition, SAHA significantly upregulated TNAP activity at a concentration of 500 nM but downregulated ENPP1 at a lower concentration of 50 nM. These results suggest that both HDACis, TSA and SAHA, downregulated ENPP1 enzyme activity and upregulated TNAP enzyme activity by decreasing ENPP1 protein expression and increasing TNAP protein expression, respectively.

### 2.3. HDACis Downregulated Extracellular Pyrophosphate Levels and Prevented CPP Crystal Formation

**Figure 4:**
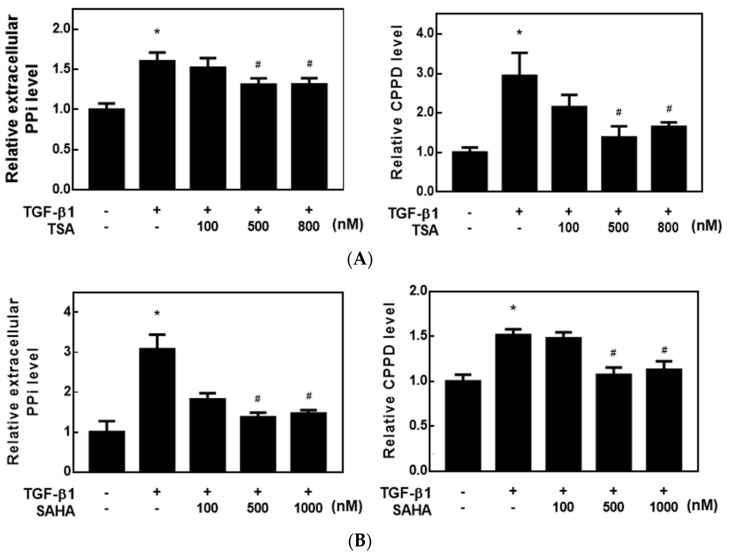
Histone deacetylase inhibitors (HDACis) decreased extracellular pyrophosphate levels and inhibited calcium pyrophosphate (CPP) crystal formation in transforming growth factor (TGF)-β1-treated chondrocytes. (**A**,**B**) Human primary chondrocytes (HC-a cells) were pretreated with different concentrations of trichostatin A (TSA) (100~800 nM) and vorinostat (SAHA) (100~1000 nM) for 30 min and then treated with 5 ng/mL TGF-β1 for (**A**) 2 and (**B**) 5 days. Cultured medium and cells were used to determine the pyrophosphate (PPi) level and CPP crystal formation, respectively. Data are expressed as the mean ± SE of at least three independent experiments. * *p* < 0.05 vs. column 1; # *p* < 0.05 vs. column 2.

Since we found that HDACis could regulate *ENPP1* and *TNAP* gene expressions and enzyme activities, we next examined whether the HDACis could decrease ePPi levels and CPP crystal formation in a cell model. As shown in Figure 4A, both TSA and SAHA significantly reduced the ePPi level in TGF-β1-treated HC-a cells. ARS dye was applied to stain calcium deposits in HC-a cells. Then, the dye was extracted, and the concentration was measured by spectrophotometry. Both TSA and SAHA significantly decreased ARS dye levels in TGF-β1-treated HC-a cells (Figure 4). The results suggest that the HDACis, TSA and SAHA, downregulated ePPi and ultimately decreased CPP crystal formation.

### 2.4. HDACis Increased the Acetylation of Histones H3 and H4

**Figure 5:**
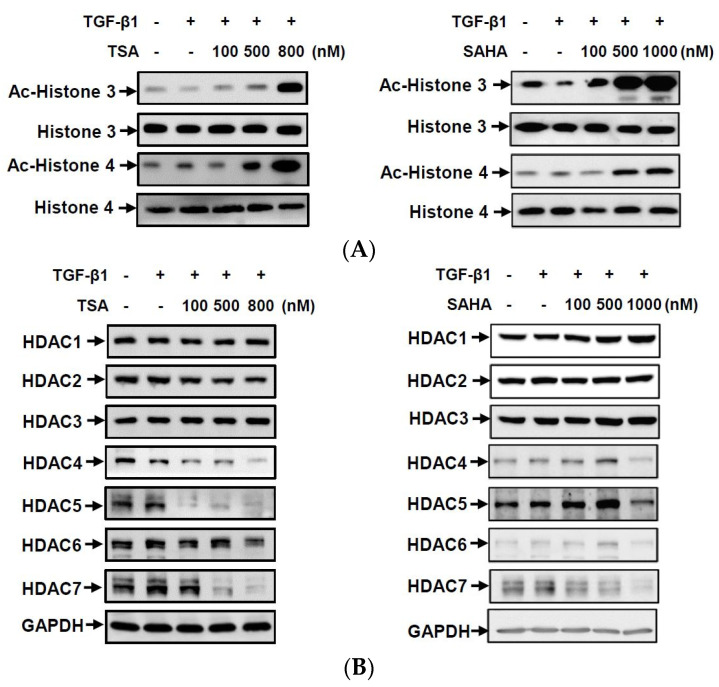
Histone deacetylase inhibitors (HDACis) downregulated class II HDAC member expressions and upregulated the acetylation of histones 3 and 4 in transforming growth factor (TGF)-β1-treated chondrocytes. (**A**,**B**) Human primary chondrocytes (HC-a cells) were pretreated with different concentrations of trichostatin A (TSA) (100~800 nM) and vorinostat (SAHA) (100–1000 nM) for 30 min and then treated with 5 ng/mL TGF-β1 for 24 h. Total cellular proteins were collected, and protein expressions were detected by Western blotting. Ac, acetylated.

**Figure 6:**
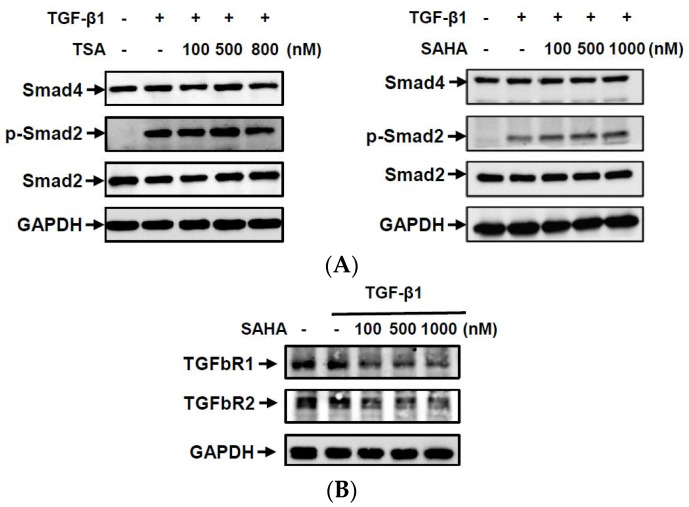
Histone deacetylase inhibitors (HDACis) did not affect Smad signal transduction in transforming growth factor (TGF)-β1-treated chondrocytes. Human primary chondrocytes (HC-a cells) were pretreated with different concentrations of trichostatin A (TSA) (100–800 nM) and vorinostat (SAHA) (100–1000 nM) for 30 min and then treated with 5 ng/mL TGF-β1 for (**A**) 2 h and (**B**) 24 h. Total cellular proteins were collected, and protein expressions were detected by Western blotting.

To understand whether the HDACis could increase levels of histone acetylation and change CPP formation-related gene expressions, we examined acetylation levels of histones H3 and H4 and protein expressions of class I and II HDACs in HC-a cells. As shown in Figure 5A, both TSA and SAHA significantly induced the acetylation of histones H3 and H4 in dose-dependent manners. In addition, neither TSA nor SAHA changed the protein expressions of class I HDACs (HDAC1, 2, and 3), but both dose-dependently decreased HDAC7 expression (Figure 5B). However, TSA and SAHA had inconsistent effects on HDAC4, 5, and 7 protein expressions. TSA dose-dependently decreased these HDAC expressions, but SAHA slightly increased their expressions in the concentration range of 100–500 nM. As it is known that ANKH and ENPP1 can be induced by TGF-β1, we next examined whether the HDACis regulated CPP formation-related gene expressions by blocking the TGF-β1 signal pathway. Smad 2 phosphorylation and Smad 4 expression are major mediators in the TGF-β1 canonical pathway. However, neither TSA nor SAHA inhibited Smad 2 phosphorylation or Smad 4 expression (Figure 6). The results suggest that the increases in histone acetylation by TSA and SAHA might be mediated through inhibition of HDAC enzyme activities and decreases in some HDAC protein expressions in a Smad-independent manner.

## 3. Discussion

TGF-β1 is known to stimulate high ePPi production by increasing expressions of ANKH and ENPP1 in chondrocytes [37,38], but it reduces TNAP expression in human pulp cells [39]. In this study, we found that TGF-β1 markedly induced mRNA expressions of *ANKH* and *ENPP1* but caused no significant change in *TNAP* mRNA expression in human primary cultured chondrocytes. These results are consistent with those reported in earlier research that used rat primary cultured chondrocytes [37]. When TGF-β1 binds to its receptor, the receptor complex activates Smad transcription factors and non-Smad molecules, such as extracellular signal-regulated kinase (ERK). However, TSA and SAHA did not change the phosphorylation level of Smad-2 (Figure 6A) or ERK (data not shown), indicating that the HDACis were unable to affect signal transduction induced by TGF-β1. Interestingly, TSA and SAHA significantly decreased TGF-βRI and TGF-βRII receptor expressions. These results suggest that HDACis might modulate expressions of ANKH, ENPP1, TNAP, and TGF-β receptors at the level of epigenetic regulation, such as the acetylation status. However more experiments are needed to confirm whether HDACis change these gene expressions by increasing histone acetylation. Mechanical loading is known to change the transient and long-term metabolism of chondrocytes and plays an important role in cartilage integrity [40,41]. A previous study in an experimental model of porcine chondrocytes showed that ATP released by mechanical loading is the major source of pyrophosphate [42]. Mechanical strain increases the expression of ANK, ENPP1, and TGF-β1 in rat chondrocytes [43]. In this study, we did not use the mechanical loading model, but HDACis reduced the gene expression of *ANKH* and *ENPP1* (Figure 1 and Figure 2). Therefore we speculated that HDACis might also reduce mechanical-loading-induced pyrophosphate.

Previous studies showed that ANKH, ENPP1, and TNAP can regulate ePPi levels, among which ANKH is the most important regulator. In addition to exporting PPi, ANKH is responsible for exporting ATP. High levels of *ANKH* mRNA were found in chondrocytes and cartilage extracts of CPPD patients [44]. *ANKH* gene mutations or polymorphisms were also related to CPPD [13,45]. High ePPi levels also contribute to bone hypermineralization, which is associated with OA disease. Calcium-containing crystals, including CPP, are common in joints and cartilage of OA patients [46], and their presence is related to OA severity [47]. Meniscal cells obtained from OA patients are easy to calcify, and calcification-related genes, such as *ENPP1* and *ANKH*, also increase [48]. However, whether calcium-containing crystals cause OA remains to be studied in depth.

HDACs are involved in the regulation of chondrocyte proliferation, maturation, and hypertrophy and are related to protecting cartilage. In recent years, however, several studies showed that HDACs contribute to the pathogenesis of OA [49]. In the early stage of mouse bone development, HDAC3 is necessary for chondrocyte maturation, but HDAC3 also activates expressions of matrix metalloproteinase 13 (MMP13) and a disintegrin-like metalloproteinase with thrombospondin 5 (ADAMTS5) via the nuclear factor (NF)-κB pathway and aggravates OA progress [50]. A study also found that cartilage of OA patients exhibited increased HDAC1 expression, which promoted activity of the Snail 1 transcription factor. Snail 1 is known as a repressor of *collagen 2* gene expression in chondrocytes [51]. On the other hand, an HDACi exhibited protective activity in cartilage in several in vitro and in vivo models. TSA inhibited inducible nitric oxide (iNOS) and cyclooxygenase (COX)-2 expressions induced by IL-1β in chondrocytes [52] and improved OA through activation of the nuclear factor erythroid 2-related factor 2 (Nrf2) signal pathway in animals [53]. SAHA, like TSA, can also inhibit inflammation in chondrocytes. It inhibits expressions of MMPs and iNOS by inhibiting mitogen-activated protein kinase (MAPK) and NF-κB [54]. In this study, we found that both TSA and SAHA decreased extracellular ePPi levels and CPP formation in human primary cultured chondrocytes. These results suggest that the HDACis, TSA and SAHA, might block both CPP crystal formation and OA pathogenesis; therefore, HDACis could be developed as potential drugs to treat or prevent OA.

Previous studies showed that CpG island hypermethylation of gene promoter region, histone deacetylation, and histone H3K9 methylation are positively correlated to gene silencing. HDACis may remove methyl-binding proteins (MePC2) from methylated cytosines and then recruit histone acetyltransferase to re-acetylate histones. The hyperacetylation of histones may further attract DNA demethylase to remove methyl groups, which results in turning on or turning off gene expressions during this process [55]. Using CpG Island Finder (http://dbcat.cgm.ntu.edu.tw/, accessed on 19 October 2021), we found that the gene promoters of *ANKH*, *ENPP1*, and *TNAP* all possess CpG islands in the promoter region. It is possible that the HDACis, TSA and SAHA, might affect gene expressions of *ANKH*, *ENPP1*, and *TNAP* by changing patterns of methylation and histone acetylation.

Previous studies [56] and the micro (mi)RNA database of SM2miR [57] showed that HDACis can regulate gene expressions through miRNAs. On the other hand, gene expressions of *ANKH*, *ENPP1*, and *TNAP* are also regulated by many miRNAs according to the GeneCards database. For example, the SM2miR database showed that TSA might change expressions of miR-106b, miR-20a, miR-20b, miR-335, and miR-93, and the GeneCards database showed that these miRNAs might regulate expression of the *ANKH* gene. Therefore, the HDACis, TSA and SAHA, can very likely regulate *ANKH*, *ENPP1*, and *TNAP* gene expressions by regulating miRNA expressions.

Currently, there is no drug to prevent or eliminate CPP formation. SAHA has been approved by the US Food and Drug Administration (FDA) to treat cutaneous T cell lymphoma, while its treatment of other cancers is being assessed in preclinical and clinical trials. In this study, we concluded that the HDACis, TSA and SAHA, were able to decrease ePPi and CPP formation by regulating ANKH, ENPP1, and TNAP expressions in human primary cultured chondrocytes. The underlying molecular mechanisms of HDACis-mediated regulation of ANKH, ENPP1, and TNAP expression might be associated with the histone acetylation status of the promoters of these three genes but not the TGF-β/Smad pathway. HDACis have the potential to be developed into drugs to prevent CPP formation or treat CPP-related diseases in the future.

## 4. Materials and Methods

### 4.1. Reagents

Two kinds of class I and II HDACis, TSA and SAHA, were obtained from Union Biomed (Taipei, Taiwan); recombinant human TGF-β1 was obtained from R&D Systems (Minneapolis, MN, USA); the anti-ANKH and anti-α-tubulin antibodies were obtained from Novus Biologicals (Littleton, CO, USA); the class I HDAC antibody sampler kit, class II HDAC antibody sampler kit, anti-Smad2, anti-phospho-Smad2 (Ser465/467), anti-histone H3, anti-acetyl-histone H3-Lys9, and anti-acetylated-lysine (Ac-K-103) antibodies were obtained from Cell Signaling Technology (Beverly, MA, USA); the anti-ENPP1, anti-TNAP, anti-acetyl-histone H4, anti-TGF-β receptor I (TGFBR1), and anti-TGF-β receptor II (TGFBR2) antibodies were obtained from Santa Cruz Biotechnology (Santa Cruz, CA, USA); and the anti-glyceraldehyde 3-phosphate dehydrogenase (GAPDH), anti-mouse immunoglobulin G (IgG) horseradish peroxidase (HRP), and anti-rabbit IgG HRP antibodies were obtained from GeneTex International (Hsinchu City, Taiwan).

### 4.2. Cell Culture

Human primary cultured articular chondrocytes (HC-a cells) were purchased from ScienCell Research Laboratories (Carlsbad, CA, USA), as well as an ethical approval document to ensure that there was no ethical issue when using HC-a cells for experiments. The cells were cultured in chondrocyte medium (CM), which included 1% chondrocyte growth supplement, 5% fetal bovine serum (FBS), and 1% penicillin/streptomycin, on dishes coated with poly-l-lysine.

### 4.3. Real-Time Reverse-Transcription Polymerase Chain Reaction (RT-PCR)

Total RNA was isolated with a PureLink RNA Mini Kit (Invitrogen, Taipei, Taiwan), and complementary (c)DNA was synthesized using a high-capacity cDNA reverse-transcription kit (Applied Biosystems, Taipei, Taiwan). Primer sequences of specific genes for the real-time PCR were: *ANKH* forward 5′-GCAGCCCACATCAAGAAGTT-3′ and reverse 5′-TCCAAAACATCACGAAACAGA-3′; *ENPP1* forward 5′-AGACAAGATGGGCATGGAAC-3′ and reverse 5′-AAAAGTGAAGGGGGTAACAGC-3′; *TNAP* forward 5′-CCTGCCTTACTAACTCCTTAGTGC-3′ and reverse 5′-CGTTGGTGTTGAGCTTCTGA-3′; and GAPDH forward 5′-ATGACATCAAGAAGGTGGTG-3′ and reverse 5′-CATACCAGGAAATGAGCTTG-3′. Each real-time PCR used 2 ng of cDNA, 200 nM of forward and reverse primers, and 10 μL of Fast SYBR Green Master Mix (Applied Biosystems) to a final volume 20 μL. The real-time PCRs were carried out on a StepOne real-time PCR system (Applied Biosystems). The real-time PCR conditions were 95 °C pre-incubation for 30 s; 40 cycles of 95 °C for 3 s and 60 °C for 30 s for amplification; and 95 °C for 15 s, 60 °C for 1 min, and 95 °C for 15 s for melting curve analysis. Messenger (m)RNA levels were calculated by the comparative CT method, for which the CT value of mRNA was normalized to GAPDH [58].

### 4.4. Western Blot Analysis

Total cellular proteins (20–50 μg) were heated to 100 °C for 5 min with a quarter volume of Laemmli buffer (4% sodium dodecyl sulfate (SDS), 20% glycerol, 10% 2-mercaptoethanol, 0.004% bromophenol blue, and 0.125 M Tris HCl at pH 6.8), separated by 10% SDS-polyacrylamide gel electrophoresis (PAGE), and then transferred to polyvinylidene fluoride (PVDF) membranes (GE Healthcare Life Sciences, Taipei, Taiwan). The PVDF membranes were blocked in blocking buffer (5% (*w*/*v*) skimmed milk powder in phosphate-buffered saline (PBS)/0.1% Tween 20) and probed overnight with various primary antibodies, then with a second antibody conjugated with HRP. The specific protein bands were visualized with an ECL Western blotting substrate (Thermo Fisher Scientific, Taipei, Taiwan) in an ImageQuant LAS 4000 biomolecular imager (GE Healthcare Life Sciences, Marlborough, MA, USA) [59].

### 4.5. ENPP1 and TNAP Colorimetric Activity Assays

HC-a cells were collected and dissolved in a buffer containing 1% Triton X-100, 1.6 mM MgCl_2_, and 0.2 M Tris Base (pH 8.1). An equal amount of cell lysate was added to 5 mM of artificial substrates: thymidine 5′-monophosphate p-nitrophenyl ester (p-nitrophenyl-TMP, p-NpTMP) (Sigma, St Louis, MO, USA) for ENPP1 activity; and p-nitrophenyl phosphate (p-NPP) (MedChemExpress, Monmouth Junction, NJ, USA) for TNAP activity [37]. The reactions were performed on a SpectraMax iD3 multi-mode microplate reader (Molecular Devices, San Jose, CA, USA), and the absorbance was measured at 410 nm every 10 min for 2.5 h.

### 4.6. Extracellular Pyrophosphate (ePPi) Assay

Culture medium was collected, and the ePPi level was detected using a PPi assay kit (BioVision, Milpitas, CA, USA) according to the manufacturer’s instructions. The fluorescence signal was quantified using a SpectraMax iD3 multi-mode microplate reader (Molecular Devices) at an excitation wavelength of 535 nm and emission wavelength of 587 nm.

### 4.7. CPP Crystal Staining Assay

High-density HC-a cells were seeded on 96-well plates at 10^5^ cells/well. Cells were treated with increasing concentrations of TSA or SAHA for 30 min and then treated with 5 ng/mL TGF-β1 for 5 days. Cells were fixed in 10% formalin and stained with 40 mM Alizarin Red S (ARS) dye (pH 4.1) for calcium mineral deposition. After extensive washing with water, the remaining dye was extracted with 10% acetic acid and quantified by a SpectraMax iD3 multi-mode microplate reader (Molecular Devices) at 405 nm absorbance [60].

### 4.8. Statistical Analysis

Experimental data were evaluated by a one-way analysis of variance (ANOVA) followed by Tukey’s post hoc test (Prism 7.0a, GraphPad Software San Diego, CA, USA). Data are presented as the mean ± standard error (SE) of at least three independent experiments. *p* < 0.05 was considered statistically significant.

## Data Availability

The data presented in this study are available on request from the corresponding author.
